# Epileptic Seizure Detection and Experimental Treatment: A Review

**DOI:** 10.3389/fneur.2020.00701

**Published:** 2020-07-21

**Authors:** Taeho Kim, Phuc Nguyen, Nhat Pham, Nam Bui, Hoang Truong, Sangtae Ha, Tam Vu

**Affiliations:** ^1^Department of Computer Science, University of Colorado, Boulder, CO, United States; ^2^Department of Computer Science and Engineering, University of Texas at Arlington, Arlington, TX, United States; ^3^Department of Computer Science, University of Oxford, Oxford, United Kingdom

**Keywords:** seizure detection, biosignal processing, biosignal classification, brain stimulation, EEG

## Abstract

One-fourths of the patients have medication-resistant seizures and require seizure detection and treatment continuously to cope with sudden seizures. Seizures can be detected by monitoring the brain and muscle activities, heart rate, oxygen level, artificial sounds, or visual signatures through EEG, EMG, ECG, motion, or audio/video recording on the human head and body. In this article, we first discuss recent advances in seizure sensing, signal processing, time- or frequency-domain analysis, and classification algorithms to detect and classify seizure stages. Then, we show a strong potential of applying recent advancements in non-invasive brain stimulation technology to treat seizures. In particular, we explain the fundamentals of brain stimulation approaches, including (1) transcranial magnetic stimulation (TMS), (2) transcranial direct current stimulation (tDCS), (3) transcranial focused ultrasound stimulation (tFUS), and how to use them to treat seizures. Through this review, we intend to provide a broad view of both recent seizure diagnoses and treatments. Such knowledge would help fresh and experienced researchers to capture the advancements in sensing, detection, classification, and treatment seizures. Last but not least, we provide potential research directions that would attract seizure researchers/engineers in the field.

## 1. Introduction

Epileptic seizure is a transient occurrence of signs or symptoms due to abnormal excessive or synchronous neuronal activity in the brain (1). Currently, about 2.3 million adults and more than 450,000 children and adolescents in the United States live with epilepsy. About 150,000 people are diagnosed with epileptic seizures each year (2). Epileptic seizures all start in the brain with sudden abnormal electrical discharges^1^. Among patients with epileptic seizures, two-thirds can control seizures through anti-epileptic medication, and another 8-10% could benefit from surgery. The remaining 25% have medication-resistant epileptic seizures and experience sudden seizure symptoms (3). Therefore, it is essential to notify the patient's medication-resistant epileptic seizure to the caretaker and analyze the pattern of related signals before, during, and after the seizure onset.

This article contributes to organizing seizure detection, classification, and treatment. We also provide potential research directions that would attract seizure researchers/engineers in the field. The existing seizure surveys reviewed seizure detection (4), classification (5–8), or treatment (9, 10). This paper discusses state-of-the-art techniques for (1) capturing the physiology signals of seizures, (2) detecting and classifying types of seizures, (3) seizures therapy, and (4) the challenges and potential seizure-related research directions.

First, accurately and reliably capturing physiology signals related to seizure is a critical step for designing robust seizure detection systems. Monitoring brain activity signal (e.g., Electroencephalogram, EEG) is the most common method to detect seizures. The EEG recording of patients with epileptic seizures has two categories of abnormal activity: interictal, abnormal signals recorded between epileptic seizures, and ictal, the activity recorded during an epileptic seizure (6). We focused on epileptic seizure detection and considered interictal and ictal EEG signals except postictal state to detect abnormal EEG signals. The EEG signature of an inter-ictal activity is occasional transient waveforms, while that of an ictal activity is composed of a continuous discharge of polymorphic waveforms of variable amplitude and frequency (11). There are two kinds of traditional EEG recording techniques: Invasive EEG and scalp EEG. The invasive EEG recording is necessary to do surgery to implant the electrodes in the brain. In the case of the scalp EEG, the user is required to attach multiple electrodes that are connecting to a monitoring device through many wires. Therefore, Patients have to suffer the inconvenience of inserting something into the body or attaching multiple electrodes. Also, for the scalp EEG, a trained physician does such a complicated setup, and the studies are often conducted in hospitals. Besides the traditional EEG-based approach, epileptic seizures can also be detected through eye (lid) movement, heart rate, blood pressure, arterial oxygenation (*SpO*_2_), respiration, sweating, and so on (4). These activities can be captured from physiology signals, including Electrooculography (EOG), electrocardiography (ECG), electromyography (EMG), electrodermal activity (EDA), motion, audio/video recording, and multimodality sensing approaches (4, 7).

We also discuss in detail the key components of these state of the art systems to provide a detailed picture of recent efforts on extracting these physiological signals for seizure detection. These systems often include some essential components as following: (1) signal acquisition, (2) signal processing, (3) feature extraction. The signal acquisition component is designed to capture physiological signals that are directly or indirectly related to seizures (4, 12). These signals often contain a lot of noises, which will be processed further using novel, yet complex algorithms to extract the signal of interests (13, 14). Next, many recent efforts have focused on building a stable setup features representing the presence of seizures to improve the detection accuracy (15–18). Hybrid time-frequency analysis features are often used to overcome the impact of human motion artifacts as well as to improve the system sensitivities (19–21). Specifically, wavelet transform analysis (WT) approaches are employed (22) to provide detailed resolutions of the seizure-related signatures on both time and frequency domains (23).

Second, after capturing the physiology signals, it is important to accurately detect and classify the type of detected seizures (5, 6, 24). Existing seizure classification methods primarily include classical machine learning approaches [e.g., support vector machine (SVM)] and novel deep-learning solutions [e.g., artificial neural network (ANN) (7)]. SVM divides data belonging to two groups into a hyperplane (25, 26). The original SVM is a binary classification, whereas the class for seizure is divided into at least three (focal seizure, generalized seizure, and healthy). State of the art SVM-approaches only can classify two classes of seizures (seizure vs. non-seizure) with high accuracy (27, 28). It is not sufficient for seizure classification. Multiclass SVM methods have been used by splitting one multiclass problem into several binary classification problems (29, 30). Although many related works have used multiclass SVM to classify various seizure types, it is impractical due to the low classification accuracy and many false alarms (29, 31, 32). Many recent efforts have focused on developing more complex learning algorithms. Especially, deep-learning solutions to detect a variety of seizures attract much attention from researchers (33). The classification performance depends on how the system structures hidden layers, such as multilayer perceptron neural network (MLPNN), adaptive neuro-fuzzy inference system (ANFIS), radial basis function neural network (RBFNN), convolutional neural network (CNN), and recurrent neural network (RNN) (34). ANN is the preferred method over SVM because it is not affected by the number of classes.

Third, after detecting and classifying different types of seizures, treatment methods need to be developed to reduce or remove the impact of seizures on patients' normal life. Even though it is difficult to find existing works in this direction, we believe that these can be done by exploring the uses of state of the art brain stimulation technique. We also discuss how the recent development in brain stimulation and interventions would help to treat seizures, such as decreasing cortical excitability with low-frequency magnetic stimulation (35) or counterbalancing the neuronal hyper-excitation through electric neural modulation (36). In particular, brain stimulation has been noted as an alternative to drug therapy to decrease the frequency of seizure or reduce the symptom. It is mostly divided into invasive and non-invasive. Although the invasive brain stimulation stimulates the problematic seizure part of the brain directly and provides a fast and accurate effect, it is necessary to do surgery to implant the stimulator inside the brain. It is very costly and may damage the brain during the operation. Thus, many patients are reluctant to this type of therapy. For non-invasive brain stimulation, there are two principal methods: transcranial magnetic stimulation (TMS) and transcranial direct current stimulation (tDCS) (10). TMS uses the principle of electromagnetic induction to focus induced current in the brain (37). The magnetic fields generated by TMS penetrate human tissue painlessly and induces electric currents that can depolarize neurons or their axons in the brain (38). tDCS is one of transcranial electrical stimulation (tES) and applies low-amplitude direct currents via scalp electrodes and penetrate the skull to enter the brain (37). Unlike other tES methods, tDCS delivers a sustained current (39) and can make the therapeutic effect through the sustained current. However, TMS and tDCS provide low spatial resolutions, which lead to modulate neuronal activity not only in the target but also in surrounding circuits (40). Transcranial focused ultrasound (tFUS) is emerging as a method that can complement the low degree of spatial focality of TMS and tDCS. We examine how brain stimulation can reduce seizures based on these three approaches.

Last but not least, inspiring from the recent development in seizure detection and classification method, we found that more efforts are needed to put into the following research direction to realize a complete, reliable, and low-cost seizure detection systems. First, we believe that the state-of-the-art seizure detection system performance is sufficient to build a robust and reliable wearable device that could be used for daily seizure monitoring and classification. Second, as the seizure signatures are detected and monitor, the recent brain-stimulation techniques can be used to reduce seizure. We also suggest different directions on how to build reliable and wearable seizure therapy systems. Lastly, we discuss how to build an integrated monitoring and stimulating seizure.

In the following, we first describe the state-of-the-art approach to capture physiological signals related to seizures in section 2 reliably. Next, we discuss recent efforts on building machine learning techniques to detect and classify seizures in section 3. In section 4, we discuss the different approaches to seizure therapy. Lastly, we summarize the overall contents of this article and provide the prospect of future research.

## 2. Analyzing Physiology Signals of Epileptic Seizure

**Figure 1:**
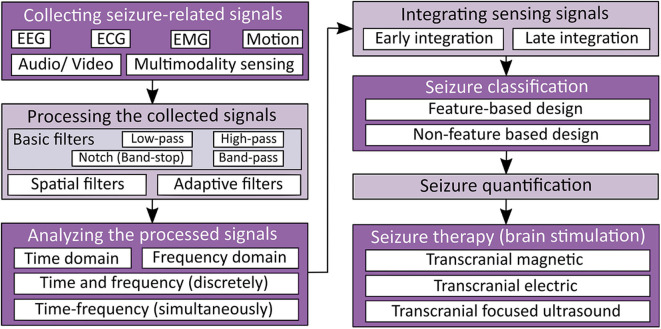
Seizure detection and therapy overview.

Seizure detection and therapy systems generally consist of five processes: (1) signal acquisition, (2) signal processing, (3) feature extraction, (4) classification, and (5) therapy (5, 6, 24). The processes mentioned above are illustrated in Figure 1. In this section, we discuss the needed signal processing steps to analyze the captured physiology signals of epileptic seizures. Upon the processed data, detection and classification algorithms could be built.

### 2.1. Collecting Seizure-Related Signals

A seizure can be detected by monitoring various physiological signals from the human body through (1) EEG, (2) EMG, (3) ECG, (4) motion, and (5) audio/video recording (4, 7). Among these physiological signals, EEG is the most popular choice because of its advantages, such as (1) the ability to capture the neural activation of the brain, (2) high temporal, and (3) spatial resolutions. However, the main limitation of traditional EEG measurement lies in its obtrusiveness and complicated setup, so it can only be performed in a controlled environment by a specialized technician. Also, some kinds of seizures like generalized onset motor seizures can be detected more clearly by measuring body movements or other physiological signals (18, 41). Thus, researchers have developed seizure detection devices using various non-EEG signals as well as EEG signals (17, 18). In the following discussion, we discuss how these recorded signals are used to detect seizure events by dividing into EEG and non-EEG methods.

#### 2.1.1. Electroencephalogram (EEG)-Based Approach

**Figure 2:**
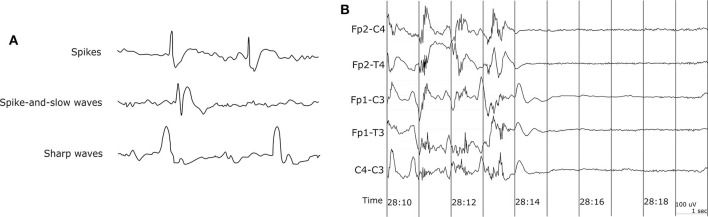
EEG waveform. **(A)** EEG waveform with paroxysmal abnormality. **(B)** Sudden death in epilepsy recorded in ambulatory EEG (42).

EEG recording is the most common method to get the biosignals for seizure detection. It measures the electrical activity of the brain. Since epileptic seizure activities appear as abnormal signal patterns on the EEG, we can use the EEG signal variation to detect seizures. EEG signals with paroxysmal abnormality show spikes, spike-and-slow waves, and sharp waves in Figure 2A. Spikes are the primary form, and their time length is 20–70 ms. The spike-and-slow waves appear after spike-wave, and their time length is 200–500 ms. Sharp waves are similar to spike-wave, but their time length is 70–200 ms (5).

The EEG recordings of patients with epileptic seizures show two categories of abnormal activity. Interictal has the abnormal signals recorded between epileptic seizures, and ictal is the activity recorded during an epileptic seizure (6). We focused on epileptic seizure detection and considered interictal and ictal EEG signals except postictal state to detect abnormal EEG signals. The EEG signature of an inter-ictal activity is occasional transient waveforms, while that of an ictal activity is composed of a continuous discharge of polymorphic waveforms of variable amplitude and frequency (11).

Many studies have been carried out for seizure detection using scalp EEG. Among them, we have selected and summarized some studies from past to recent which clearly explained the seizure detection procedure, as shown in Table 1. Attaching EEG electrodes on all parts of the scalp is reasonable because there are many types of seizures, and the initial location which was generated the abnormal EEG signal is different. However, it causes mobility impairment, increases the cost of the measuring device, and is inappropriate for patients who need continuous seizure monitoring.

**Table 1:** Seizure detection depending on the signal types.

**References**	**Signal acquisition^**a**^**	**Seizure type^**b**^ (43)**	**Noise filtering**	**Feature extraction^**c**^**	**Experiment**	**Results^**d**^**
(44)	Scalp EEG	Focal onset seizures and GTCS	High-pass filter Low-pass filter	Time-frequency domain features - WT	652 h of scalp EEG, including 126 seizures in 28 patients	SEN 76%, false detection rate 0.34/h, median detection delay 10 s
(45)	Scalp EEG	Focal onset seizures	Band-pass filter	Time-frequency domain features - WT	The algorithm was tested on scalp EEG recordings from 14 patients, totaling 75.8 h with 63 seizures	SEN 90.5%, false detection rate 0.51/h, median detection delay 7 s
(46)	Scalp EEG	Unspecified	Band-pass filter Butterworth filter	Frequency-domain features—stationary WT	Scalp EEG records of 24–48 h of duration of 18 epileptic patients	SEN 87.5% and SPE 99.9%
(47)	Scalp EEG	Focal seizures	Band-pass filter	Time-frequency domain features - WT	10 participants' hospital archived 127-h EEG recordings with 310 ictal discharges	SEN 93% and precision 55%
(24)	Ear EEG	FIAS	Band-pass filter spatial filter	Time and frequency features	In twelve with focal onset impaired aware, four additional electrodes were glued on the skin behind the ears	With scalp EEG, detection had a median SEN of 100% and a FDR of 1.14 per hour. With behind-the-ear EEG, it had a median SEN of 94.5% and a FDR of 0.52 per hour
(48)	ECG	Unspecified neonatal seizures	A central moving average filter	Time and frequency features	The performance was evaluated on a large dataset of 208 h from 14 newborn infants	SEN 60% and SPE 60%
(49)	ECG	Focal myoclonic and GTCS	Unspecified	Unspecified	Three epilepsy patients are admitted in their facilities for 14, 13, and 9 nights, respectively. The EMFIT bed sensor is used to monitor the heart rate variability	SEN 75% and SPE 70.4%
(50)	ECG	Focal onset and generalized onset seizures	Unspecified	Time-domain features	Single-lead ECG signals were recorded from patients suffering from focal and generalized seizures. Two algorithms are proposed: one quantifies changes in the QRS morphology using PCA, and one assesses cardiorespiratory interactions using phase rectified signal averaging	PPV of 86.6 and 77.5% and SEN of 100 and 90% were achieved for focal and generalized seizures respectively
(51)	ECG	FTCS and GTCS	Median filter	Time-domain features	126 seizures from 43 patients were recognized. The best-performing HRV algorithm combined a measure of sympathetic activity with a measure of how quickly HR changes occurred	SEN 93.1% for all seizures and 90.5% for nonconvulsive seizures, FDR 1.0/24 h, and PPV 87%
(52)	PPG and ECG	Focal onset seizures	Notch filter Low-pass filter Band-pass filter Butterworth filter	Time-domain features	The test was applied to recordings of 11 patients in a hospital setting with 701 h capturing 47 TLE	The SEN of the hospital system, the wearable ECG device and the wearable PPG device were respectively 57, 70, and 32%, with corresponding FDR per hour of 1.92, 2.11, and 1.80
(18)	surface EMG and ECG	Tonic seizures and GTCS	High-pass filter Anti-aliasing filter Butterworth filter Notch filter	Time and frequency features	Six patients with tonic seizures were included	The best performance in this study is SEN = 0.53 and FDR = 1.49 or SEN = 0.63 and FDR = 4.03, depending on the choice of parameters
(53)	surface EMG	GTCS	Notch filter Band-pass filter	Time and frequency features	In 33 patients, 1,399 h of surface EMG data were recorded, averaging 42 (*SD*±17) h per patient. Eleven patients had 21 GTCS recorded by video-EEG	20 of 21 GTCS were detected (95% SEN, 95% confidence interval 76–100) all within 60 s (mean 15.2 s range -4 to 56 s)
(41)	ACM	Tonic seizures	Low-pass filter	Time-domain features	Three experts divided the corresponding ACM-signals into classes using video and accelerometric information	For off-line, 80% of the tonic seizures were detected with a positive predictive value of 0.35, and 42% of the false positives is also a seizure
(54)	ACM	Hypermotor seizures (GTCS)	Unspecified	Time-domain features	7 patients between 5 and 15 years old and 51 hypermotor seizures	SEN 95.71% and PPV 57.84%
(55)	ACM	FTCS	Unspecified	Time-domain features	Thirty-nine FTCS were recorded in 20 patients	The wireless wrist accelerometer correctly detected 35 seizures. The mean SEN per patient was 91% (95% confidence interval 80–100)
(56)	Audio	GTCS and long generalized tonic seizures	Unspecified	Unspecified	Ten patients with symptomatic generalized or multifocal epilepsies take participate in the experiment	SEN 0.81 (range: 0.33–1.00) and PPV 0.40 (range: 0.06–1.00)
(57)	Audio	GTCS	Unspecified	Unspecified	166 audio clips of 30 s duration from 83 patients with one clip during a seizure period and one clip during a non-seizure control period for each patient	PPV 0.91
(58)	Image processing	Focal clonic seizures of newborns	A differential filtering	Unspecified	They extracted the average motion signal from a video of a newborn affected by a neonatal seizure	Single window processing has SEN 0.93 and SPE 0.67, while Three windows processing has SEN 0.60 and SPE 0.86
(59)	EDA + ACM	FTCS	Unspecified	Time and frequency features	A total of 16 FTCS were recorded from seven patients	The system detected 94% of the FTCS with 0.74 per 24 h FDR
(16)	EDA + ACM	GTCS	Unspecified	Time and frequency features	The 9 patients' recordings included 20 GTCS over a total of 738 h	SEN 95% and FDR 0.48
(60)	EDA + ACM	Unspecified (including seizures without motor activity)	Low-pass filter	Time-domain features	EDA and ACM from 8 patients were analyzed. Different types of seizures, including seizures without motor activity, were taken into account	Overall SEN 89.1 and SPE 93.1% For seizures without motor activity, SEN 97.1% and SPE 92.9%
(17)	EDA + ACM + temperature + HR + *SpO*_2_	FIAS, FTCS, and GTCS	A 3-s smoothing filter	Time-domain features	339 h of data (26 seizures) collected from 10 patients in an epilepsy monitoring unit	100% sensitivity and high accuracy in six out of 10 patients
(61)	EEG-video-audio	Focal onset seizures	Low-pass filter	Unspecified	12 patients with focal onset seizures had undergone 24 h EEG-video-audio monitoring over 1–15 days (mean 10.5, standard deviation 3.86)	SEN 81.42% and FDR 5.38/h

a *ACM, Accelerometry; HR, Heart rate*.

b *FIAS, Focal onset impaired awareness seizures; FTCS, Focal to bilateral tonic-clonic seizures; GTCS, Generalized tonic-clonic seizures*.

c *WT, Wavelet transform*;

d *PPV, positive predictive values; SEN, sensitivity; SPE, specificity; FDR, False Detection Rate*.

**Figure 3:**
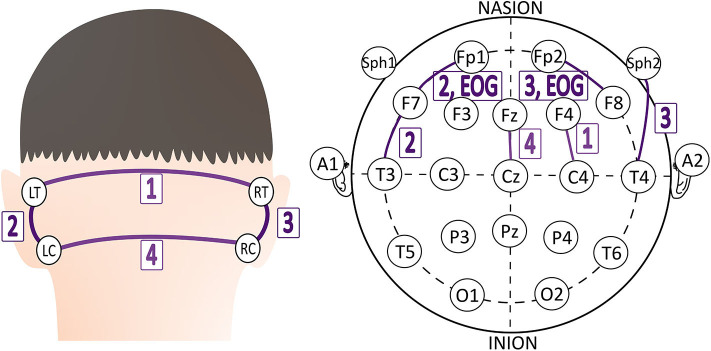
The best match-up scalp EEG channel of each behind-the-ear EEG channel on 12 patients (24).

Capturing EEG signals around the ear is a promising finding which can minimize the obtrusiveness of conventional EEG methods. The evoked responses from the ear-EEG are typically 10–20 dB lower in amplitude than those of traditional scalp EEG recordings while maintaining a similar signal-to-noise ratio (SNR) (62). Mikkelsen et al. (63) compared 32 conventional scalp electrodes with 12 ear electrodes. The measured signal from the ear electrodes reflects the same cortical activity as that from nearby scalp electrodes. Bleichner et al. (63) also worked for the comparison between a traditional EEG cap setup and their around-the-ear electrode array (cEEGrid). They have shown that their system can capture meaningful EEG signals such as eye-closing alpha wave, sleep spindles, and epileptic spike-wave. Gu et al. (24) utilized the cross-head and unilateral channels from the behind-the-ear EEG. Temporal waveform and frequency content during seizures from behind-the-ear EEG visually resembled those from scalp EEG. Especially, this paper provides the coherence between the behind-the-ear EEG channel and the best match-up scalp EEG channel on 12 patients like Figure 3. McLean et al. (42) reported the sudden death in epilepsy recorded in ambulatory EEG. In Figure 2B, the seizure activity abruptly terminated, and the EEG became a flat line. The EEG variation graph of Figure 2B shows that these EEG channels may have significant patterns for detecting a seizure. Also, the EOG from LT-LC and RT-RC have similar morphology to that from Fp1-F7 and Fp2-F8, respectively.

#### 2.1.2. ECG, EMG, Motion, Audio, and Video-Based Approach

EEG-based measurement usually implies that the sensors need to be attached to a human head for seizure defections. Also, EEG monitoring is prone to errors in interpreting complex signals of EEG and is mainly used to detect seizures from temporal lobe epilepsies (2, 64). Therefore, researchers have developed seizure detection devices with various other methods. Among the relatively recent studies, we tried to select papers, which use other signals more actively than EEG and follow the clear seizure detection procedure, as shown in Table 1. For example, contactless sensing devices such as mattress sensor [Emfit^2^, MP5^3^], carry-on devices such as smartwatches or wrist devices [Cogan (65), Embrace (59), Inspyre^4^], smart textiles (Neuronaute^5^), and temporary tattoos (4) can be used to detect epilepsy. We found that ECG, EMG, motion, and audio/video recording approaches have been used to monitor epilepsy.

*Electrocardiography (ECG)* monitoring measures the electrical properties of the heart and detects heart rate (HR) and heart rate variability (HRV). Most of the generalized tonic-clonic seizures (GTCS) cause an increase in HR (66). Such events subsequently increase the risk of sudden unexpected death in epilepsy (SUDEP) (42). HRV is also useful to distinguish focal seizures with physical exercise (51). The most common pattern of HRV associated with focal onset impaired awareness seizures is an initial steep acceleration at the onset of the seizure (67). The HRV in temporal lobe seizures is different from that in psychogenic non-epileptic seizures (68). ECG can be used to detect a seizure. However, the accuracy and ability to detect a seizure early are still very limited.

*Electromyography (EMG)* monitoring measures electrical activity in response to a nerve's stimulation of the muscle. *Motion* is detected using accelerometers measuring the accelerations of objects in motion along reference axes (69). Both signals could be useful to detect generalized onset motor seizures.

For *Audio/video recording*, Arends et al. (56) evaluated the performance of audio-based detection of primary seizures (tonic-clonic and long generalized tonic). They adapted the sound threshold by training during the first week. Recognizable sounds over the threshold occurred in 23 of the 45 significant seizures. This result signifies the use of only audio recording has definite limitations. Ntonfo et al. (58) proposed the image processing approach to detect the focal clonic seizures of newborns, which are related to the periodic movements of parts of the body. They extracted an average luminance signal representative of the body movements from a video of a newborn. Single window processing has high sensitivity ($\frac{TP}{TP+FN}$, where *TP*: True Positive, *FN*: False Negative) and low specificity, ($\frac{TN}{TN+FP}$, where *TN*: True Negative, *FP*: False Positive), while multiple interlaced window processing has low sensitivity and high specificity. It is necessary to apply the advanced window protocol to improve performance.

#### 2.1.3. Multimodality Sensing Approach

The multimodality sensing approach may improve sensitivity and lower false-positive alarms by combining the profits of each sensor, like sensing EMG signals for tonic seizure detection (12). We have chosen a number of multimodality sensing studies for the purpose of dealing more with studies that use other signals more actively than EEG, as shown in Table 1. Electrodermal activity (EDA) refers to the variation of the electrical properties of the skin in response to sweat secretion (70). EDA is mainly used with other sensors to detect seizures, especially with ACM (59).

Cogan et al. (65) detected epileptic seizures using wrist-worn bio-sensors, which detect heart rate (HR), arterial oxygenation (*SpO*_2_), ACM, EDA, and temperature. They observed the seizure pattern of *HR ↑*⇒ *SpO*_2_ ↓⇒ *EDA ↑*. Using EEG and non-EEG signals together could be more appropriate to employ the seizure detection system precisely and extensively. Pauri et al. (61) applied EEG-video-audio monitoring to 12 patients with refractory focal seizures using 15-channel EEGs (video-cassettes). Greene et al. (71) combined EEG monitoring with ECG monitoring simultaneously for the robust detection of neonatal seizures.

### 2.2. Processing the Collected Signals

To collect signals, we can use wet electrodes or dry electrodes. Although the use of dry electrodes is suitable for continuous signal collection, we still need to rely on the conductive paste and gripping force of the earpieces to address the gap between the electrodes and the user's skin (72). Therefore, wet electrodes are used to maintain signal quality. And gold-plated copper electrodes are proper material due to the resistance to skin oil and sweat and rare skin allergy (73). Signal processing is necessary to get the clear biosignal waveform in the most significant frequency range (1–35 Hz) without signal distortion. Raw signal is influenced by noises from power-line and other equipment, and the signal is a mixture of several biological signals, including EEG, EOG, and EMG signals. Thus, we need to use filters.

#### 2.2.1. Basic Filters

Typical four types of the basic filter have been used to get the clear biosignal: a low-pass, high-pass, band-pass, and notch (= band-stop) filter. In the United States, the notch filter is set at 60 Hz^6^ because the 60 Hz power-line frequency noise from wires, light fluorescent, and other equipment can contaminate biosignal records. The high-pass filter can remove the low-frequency artifacts noise due to poor contact state of electrodes or the sweat of the patient under the electrodes. Furthermore, the median filter can reduce noise and high-frequency oscillations in signal data (74). However, these filters do not preserve all designated frequencies and cannot extract the specific biosignal among the overlapped biosignals spectrum (75). For example, EOG signals by eye movements or blinks propagate to the scalp electrodes creating noises in the recorded EEG signals.

#### 2.2.2. Spatial Filters

The spatial filter technique, such as Independent Components Analysis (ICA), is a promising solution to solve the challenge of overlapped artifacts in EEG recording. Jung et al. (76) applied the spatial filters derived by ICA, which can separate and remove ocular artifacts from the recorded EEG signals. ICA technique, however, requires the use of multiple EEG electrodes to provide spatial information with the captured signals. In other words, ICA can decompose the independent components only when the number of data channels is more than that of signal sources (77). Also, ICA does not work when the training data set is too small (76).

The regression-based technique is a proposed solution to overcome the limitation of ICA. We can apply the regression approach to any number of EEG channels. Regression-based noise filtering has two phases. First, the calibration phase determines the transfer coefficients between other biosignal channels and EEG channels. Second, the correction phase estimates the noise component in the EEG recording (78). Due to this procedure, it is challenging to apply this filter in real-time. The coefficients should be controlled in the normal range. Once the coefficient is out of the range, it is not trivial for the calibration phase to turn them over to the normal range.

The wavelet-based technique is another denoising method that has been proposed for EEG signals. The wavelet-based technique compares each wavelet coefficient to a predetermined threshold and sets it to zero if its magnitude is less than the threshold (78). This technique can work in real-time and does not require the prior data of the artifacts (79). However, choosing the threshold level is a complicated process.

Lastly, if EEG recordings have multi-channel, they give blurred images of brain activity due to the volume conduction (80). In this situation, spatial filters can improve the SNR using the common spatial pattern (CSP) algorithm. The CSP extracts new time series whose variances are optimal for the discrimination of two populations of EEG based on the simultaneous diagonalization of two covariance matrices (81). Several related works have demonstrated the performance of spatial filters for multi-channel EEG (80, 81).

#### 2.2.3. Adaptive Filters

**Figure 4:**
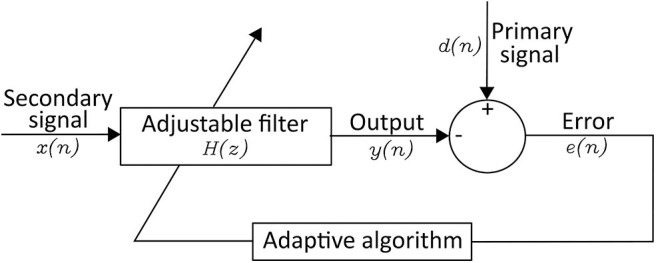
Structure of an Linear Adaptive Filter.

Adaptive filter adapts the coefficients of the filter to generate a signal similar to the noise (75). A linear adaptive filter is made up of a primary signal (= corrupted signal) *d*(*n*), a secondary signal (= reference signal) *x*(*n*), an adjustable filter *H*(*z*), an output from the adjustable filter *y*(*n*), and an error *e*(*n*) in Figure 4 (82). The adaptive filter usually adjusts the coefficients of filter to minimize the squared error between *d*(*n*) and *y*(*n*) (83). Correa et al. (84) arranged a cascade of three adaptive filters to remove multiple artifacts and got the EEG signal from EEG + artifacts (EOG, ECG, and power-line frequencies). However, the linear adaptive filter cannot deal with non-linear signals.

Researchers have developed a neural network (NN) and fuzzy network (FN) to control non-linear signals. NN is made up of an input layer, hidden layer, and output layer, and users do not know the hidden layers. Fuzzy logic analyzes analog data as logical variables having continuous values between 0 and 1 (85). Each method has the following limitation. The structure of NN is challenging to decide, and the learning efficiency of FN is lower than that of NN (86). Combining them as a fuzzy neural network (FNN) is one solution to complement each drawback. However, FNN requires the training data in advance for the backpropagation, making the real-time application difficult (87).

## 3. Classifying and Detecting Epileptic Seizure

Seizure classification mainly categorizes the input data into one of two groups: seizure and non-seizure. Under specific requirements, the group of seizures can break down into sub-categories depending on the location of the source and symptoms. Those are considered as multiclass classification. Feature-based approaches, including feature extraction and conventional machine learning techniques, have been widely adopted to identify epileptic seizures (7). For each specific data set, the studies listed in Table 2 imposes different classifier configurations and features. Although we tried to cover recent studies in Table 2, we also introduced some previously published papers to represent typical classification methods or data that were used well in the past. After the recent success of Deep Learning, many researchers start applying Deep Learning for medical problems, especially epileptic seizure detection/classification (33).

**Table 2:** Classification for seizure detection [recommended comprehensive analysis: (88, 89)].

**References**	**Classification^**a**^**	**Signal**	**Input data**	**Experiment**	**Results^**b**^**
(26)	RBF SVM	Scalp EEG	Constructing a feature vector that unifies in a single feature space the time-evolution of spectral and spatial properties of the brain's electrical activity	They trained on 2 or more seizures per patient and tested on 916 h of continuous EEG from *24 patients*	The algorithm detected 96% of *173 test seizures* with a median detection delay of 3 s and a median false detection rate of 2 false detections per 24 h period
(90)	Multiclass SVM	Scalp EEG	Fuzzy-rules-based sub-band specific features	Equal to (26)	SEN 98% and FDR 3%
(30)	Multiclass SVM	Scalp EEG	Feature extraction by computing the wavelet coefficients and the Lyapunov exponents	They used the data described in (91) (Five data sets: *each set has 5 subjects* and 2360 s duration, *only one set shows seizure activity*) and tried to discriminate the EEG signals	The total-classification accuracies of the SVM, PNN, and MLPNN were 99.28, 98.05, and 93.63%, respectively
(92)	MLPNN	Scalp EEG	Analysis with discrete wavelet transform and line length (93) feature extraction	Equal to (30)	For the classification problem between seizure and non-seizure, SEN 98.61% and SPE 94.60%
(94)	MLPNN	Scalp EEG	Maximum, entropy, average, standard deviation, and mobility from each sub-band	Equal to (30)	The classification of the EEG signals achieved with approximately 97.5% accuracy and the variance of 0.095%
(95)	ANFIS	Scalp EEG	Maximum, minimum, mean, standard deviation from each sub-band	Equal to (30)	Average SEN 98.68% and SPE 99.67% from five EEG datasets
(96)	RBFNN	Scalp EEG	Unspecified	EEG signals of *418 patients* with epilepsy are recorded using Nihon-Kohden EEG machine (*204 partial epilepsy samples, 47 primary generalized epilepsy samples*)	MLPNN: 8.8% and 19.1% error rate for focal and generalized seizure, RBFNN: 3.4% and 10.6% error rate for focal and generalized seizure
(20)	RNN	Scalp EEG	Dominant frequency, average power in the main energy zone, normalized spectral entropy, spike rhythmicity, and relative spike amplitude	They used the data described in (91) (Two data sets: *each set has 5 subjects* and 2,360 s duration, *only one set shows seizure activity*) and tried to discriminate the EEG signals	Epileptic detection accuracy 99.6% with a single input feature
(97)	RNN + RBFNN	Scalp EEG	Wavelet entropies, sample, and spectral entropies	Equal to (30)	99.75% accuracy for detecting normal vs. epileptic seizures 94.5% accuracy for detecting normal vs. interictal focal seizures
(33)	CNN	Scalp EEG	Long-term EEG signals	The total recording time of EEG was of 1124.3 h during which *97 seizures* from *24 patients*. EEG signals were converted into EEG plot images, each of which was classified by CNN as seizure or non-seizure	The median true positive rate of CNN is 74%
(98)	CNN	Scalp EEG	Spectral and temporal features from EEG epilepsy data	Seizure detection using cross-patient EEG dataset of *23 patients*. The dataset has 969 h with *173 seizures*	Overall SEN of 90.00%, SPE of 91.65%, and accuracy of 98.05%

a *RBF, Radial basis function; SVM, Support vector machine; ANN, Artificial Neural Network; MLPNN, Multilayer perceptron neural network; ANFIS, Adaptive neuro-fuzzy inference system; RBFNN, Radial basis function neural network; RNN, Recurrent neural network; CNN, Convolutional neural network*.

b *SEN, Sensitivity; SPE, Specificity*.

### 3.1. Feature-Based Design

In this protocol, the number and type of features have a significant impact on seizure detection performance. There are several feature extraction methods, including time-domain features, frequency-domain features, time and frequency features (discretely), and time-frequency domain features (simultaneously).

#### 3.1.1. Feature Extraction

##### 3.1.1.1. Time-domain analysis

Time-domain analysis works for the stationary signals, but biosignals are non-stationary. One method to quantify a non-stationary time series is to consider it as a large number of stationary segments (99). There are 12 key features in three categories: (1) *mean* and *standard deviation* for a time series with symmetric distribution; (2) *median, mode, range, first quartile*, and *third quartile* to measure the locations of a time series; (3) *maximum, minimum, variation, skewness, kurtosis* to pull out the shape characteristics of a time series (99). Besides, existing works have used slope sign change, Willison amplitude (100), Lyapunov exponents (13), and Hjorth parameters (19) to extract features from EEG signals.

##### 3.1.1.2. Frequency-domain analysis

There are three basic techniques for frequency-domain analysis: Fast Fourier transform (FFT), Eigenvector, and Autoregressive (101). FFT decomposes a function (signal) of time into a frequency component fast by rearranging the input elements in a bit-reversed order and building the decimation in time (102). Fourier transform is only suitable when we are interested in what frequency components exist, not times occurring the frequency components (23). However, the time that a specific frequency component happens is essential to analyze biosignals. To solve this problem, a short-time Fourier transform (STFT) uses the idea that some part of a non-stationary signal at any given interval of time is a stationary signal. Johnson et al. (103) extracted relative power spectral density (PSD) value for each 1 Hz bin from EEG 1–40 Hz to check the state of drowsiness.

Eigenvectors are employed to calculate the signal's frequency and power from artifact dominated measurements (101). These methods are based on an eigen decomposition of the correlation matrix of the noise-corrupted signal and produce high-resolution frequency spectra even when the SNR is low (14). There are three eigenvector methods with higher resolution (Pisarenko, MUSIC, and Minimum-Norm) (104). The Pisarenko algorithm is particularly useful for estimating a spectrum containing sharp peaks at the expected frequencies (105). The MUSIC method eliminates the effects of spurious zeros by using the averaged spectra of all of the eigenvectors corresponding to the noise subspace (106). The Minimum-Norm method puts false zeros inside the unit circle and calculates the desired noise subspace vector from the eigenvectors (107).

Autoregressive methods estimate the PSD of the EEG signal using a parametric approach. These methods solve the spectral leakage problem and yield better frequency resolution (101). Yule-Walker method may lead to incorrect parameter estimates in the case of nearly periodic signals (108). As an alternative, Burg's method first estimates the reflection coefficients, and then the parameter estimates are determined using the Levinson-Durbin algorithm (108).

##### 3.1.1.3. Time and frequency features

Using both time- and frequency-domain features can improve seizure classification performance. Srinivasan et al. (20) used three frequency-domain features (dominant frequency, average power in the primary energy zone, and normalized spectral entropy) and two time-domain features (spike rhythmicity and relative spike amplitude). Iscan et al. (109) combined time and frequency features to distinguish between seizure and healthy EEG segments. They got time-domain features using the cross-correlation method and frequency-domain features calculating the PSD.

##### 3.1.1.4. Time-frequency domain analysis

Time-frequency domain analysis studies a signal in both the time and frequency-domains simultaneously. Time-frequency distribution (TFD) and wavelet transform analysis (WT) are the principal techniques of time-frequency domain analysis.

The basic idea of TFD is to devise a joint distribution of time and frequency that describes the energy density or intensity of a signal simultaneously in time and frequency (110). In this distribution, we can calculate the fraction of energy in a specific frequency and time range, and the distribution of frequency at a particular time. It is done by constructing a joint time-frequency function with the desired attributes and then obtaining the signal that produces the distribution (110). Boashash et al. (111) performed TFD feature extraction on multi-channel recordings for seizure detection in newborn EEG signals. They selected the optimal subset of TFD features using the wrapper method with sequential forward feature selection.

WT is an alternative to STFT. STFT gives information about the spectral components at any given interval of time, but not at a specific time instant (23). It causes a problem of resolution. WT gives a variable resolution using the characteristics that high frequencies are better resolved in time-domain, and low frequencies are in frequency-domain (23). WT can capture very minute details, sudden changes, and similarities in the EEG signals (22). It is more effective than other methods because biosignals are non-stationary (112). WT transforms a small wave (a mother wavelet) as a pattern and expresses an arbitrary waveform on the scale of magnification and reduction. WT classified into continuous wavelet transform (CWT) and discrete wavelet transform (DWT). The CWT *Y*(*j, k*) is defined by the following equation for any fixed-function Ψ_*j, k*_(*t*) in Equation (1). The mother wavelet (Ψ_*j, k*_(*t*)) is shifted by a small interval of *j* in the x-axis, and correlation coefficients are computed. This procedure is repeated for various scaling factors *k* (dilations) in the y-axis (22).

(1) $$\begin{array}{l} Y\left(j,k\right)=\int f\left(t\right){\text{Ψ}}_{j,k}\left(t\right)dt \end{array}$$

CWT is computed by changing the scale of the analysis window, shifting the window in time, multiplying by the signal, and integrating overall times (23). However, the CWT has disadvantages such as severe redundancy of coefficients, difficulty in managing infinite wavelets, and lack of analytical methods that can easily calculate for most functions. The DWT solves these disadvantages by scaling and moving discretely, not continuously. The DWT employs two sets of functions called scaling functions and wavelet functions. These functions are related to the low-pass filter [*g*(*n*), the mirror vision] and high-pass filter [*h*(*n*), the discrete mother wavelet], respectively (113). In the sub-band decomposition of DWT, each stage consists of two digital filters and two downsamplers by 2. The first stage receives a signal *x*(*n*) and provides the detail *D*_1_ and the approximation *A*_1_ (114). The first approximation is further decomposed continuously. Many related works have used WT to extract features (115).

##### 3.1.1.5. Integrating sensing signals from multiple channels

Shoeb et al. (116) extracted four features (*m* = 4) representing waveform morphology on each of 21 channels (*n* = 21). Then they assembled these features into a feature vector by concatenating them orderly called the early integration (EI) architecture. They also studied the performance of a patient-specific detector with an alternative architecture called the late integration (LI) architecture. In this structure, the *m* features of each channel assembled into a distinct feature vector and are assigned to the individual class (seizure or non-seizure). LI allows for the independent classification of activity on each channel, whereas EI summarizes interrelations between channels.

##### 3.1.1.6. *Lesson learned*

Recording EEG signals is crucial because almost all seizures start from the brain. However, EEG measurement requires attaching many electrodes on the scalp with mobility impairment and making continuous measurement difficult. Therefore, we look forward to developing the devices which measure EEG signals without causing discomfort. Recording EEG signals around the ear is an emerging method to record EEG signals on the scalp. We confirmed some possibilities by comparing the similarity between EEG signal measurements around the ear and those on the scalp from many related works. Furthermore, we can get various biosignals as well as EEG around the ear (117).

The future seizure detection system is necessary to improve the signal processing procedure using spatial and adaptive filters. Basic filters do not entirely remove noise and not preserve all designated frequencies and cannot extract the specific biosignal among the overlapped biosignals spectrum (75). As we saw in Figure 3, EEG recordings have multi-channels even around the ear. Spatial filters can improve the SNR among several channels and surrounding noises using the CSP algorithm. Adaptive filters reflect the previous signal error through the self-developed adaptive algorithm. Linear or non-linear signals are controlled depending on the adaptive algorithm.

Recent papers have applied WT to the seizure detection system to analyze the processed signals. Time-domain analysis and frequency-domain analysis are easy to use and give clearly defined features. However, they may not catch the minute features for seizure because biosignals are non-stationary. Even though there are alternatives like making a large number of stationary segments from any given interval of time, they still have a problem of resolution. Meanwhile, WT can capture very minute details, sudden changes, and similarities in the EEG signals (22). WT classified into CWT and DWT. CWT has disadvantages about the redundancy of coefficients, difficulty in managing infinite wavelets, and lack of analytical methods. DWT is usually used to solve these problems. Daubechies wavelet is the most commonly used wavelet for DWT, and the interested reader for the wavelet-based EEG processing can refer to (22) for more details.

#### 3.1.2. Feature Classification Algorithms

##### 3.1.2.1. Support vector machine (SVM)

**Figure 5:**
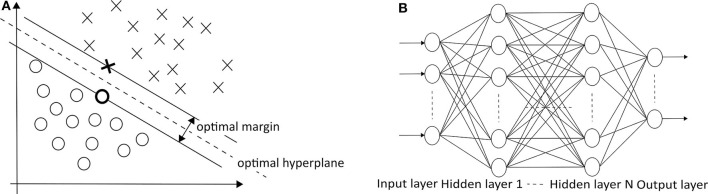
**(A)** SVM (O: positive cases, X: negative cases) (25) and **(B)** MLPNN (113) architectures.

SVM is a linear classifier that uses a hyperplane (25) to separate the data space. The mathematical expression of a hyperplane is the general form of a linear equation in multi-dimensional space.

(2) $$\begin{array}{l} a_{1}x_{1}+a_{2}x_{2}+a_{3}x_{3}+...+a_{n}x_{n}=b, \end{array}$$

which must have at least one *a*_*i*_ other than zero. Given a dataset, there could be many hyperplanes that separate the data. SVM maximizes the distance between the nearest points from each group toward the hyperplane, as described in Figure 5A. This distance is called a margin. Conventional linear SVM has a limitation due to the non-linear changes of biosignals. A non-linear SVM classifier using an RBF kernel is potentially a proper approach because the seizure and non-seizure classes are not linearly separable. This approach detected 96% of 173 test seizures in a median detection delay of 3 s (26). When the categories of seizure are divided into more than two groups (e.g., focal seizure, generalized seizure, healthy), the binary classification is not sufficient to distinguish data. In this case, the SVM method for dealing with multiclass is applied to handle the problem.

The development of multiclass SVM follows two approaches. One vs. rest approach is a method of binarizing the i-th class and the remaining *M*−1 classes. This process is repeated in the same operation for the other classes. A total of M hyperplanes are created. On the other hand, one vs. one approach is to select two of the M classes to create a hyperplane, then select the other two class combinations and repeat the same operation. A total of *M*(*M*−1)/2 hyperplanes are created. The one vs. rest approach has an imbalance in the size of the two sets, unlike the one vs. one approach. However, the one vs. rest approach is mainly used because the total number of hyperplanes increases linearly with the number of classes. Many related works have used multiclass SVM to classify seizure states (29, 30).

##### 3.1.2.2. Multilayer perceptron neural network (MLPNN)

In *MLPNNs*, each neuron in the hidden layer sums its input signals after multiplying them by each link weights and computes its output as an activation function of the sum, as shown in Figure 5B (113). The activation function can be the rectified linear unit (ReLU), hyperbolic tangent, and so on. Guo et al. (92) used Bayesian regularization back-propagation to train MLPNN, which updates the weights and biases depending on Levenberg-Marquardt optimization. It minimizes a combination of squared errors and weights and then determines the correct combination to produce a network that generalizes well. Their network structure has one input layer with five neurons, one hidden layer with 10 neurons, and one output layer with one neuron (0—the normal/non-seizure EEG, 1—the seizure EEG). Naghsh and Aghashahi imported the feature vectors into an MLPNN system to classify the signal into three states of normal (healthy), a seizure-free interval (interictal), and a full seizure interval (ictal) (94).

##### 3.1.2.3. Adaptive neuro-fuzzy inference system (ANFIS)

**Figure 6:**
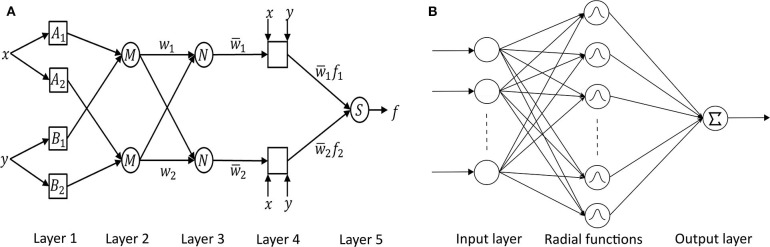
**(A)** ANFIS (118) and **(B)** RBFNN (119) architectures.

Neuro-fuzzy systems utilize the mathematical properties of ANNs in tuning rule-based fuzzy systems to approximate the way humans process information (95). Especially, *ANFIS* (118) has shown significant results in modeling non-linear functions. A type-3 ANFIS has five layers like Figure 6A. A circle and square indicate a fixed and adaptive node, respectively. In layer 1, the input values pass through the selected fuzzy membership function (μ*A*_*i*_ and μ*B*_*i*_, *i* = 1, 2). This function could be a bell-shaped with a maximum equal to 1. *Premise parameters* {*a*_*i*_, *b*_*i*_, *c*_*i*_} in the function change during the training process. In layer 2, each simple multiplier multiplies the output values of layer 1 [*w*_*i*_ = μ*A*_*i*_(*x*)μ*B*_*i*_(*y*), *i* = 1, 2]. In layer 3, each normalization function produces ${\bar{w}}_{i}=\frac{w_{i}}{w_{1}+w_{2}},i=1,2$. In layer 4, the output values of layer 3 go into the Takagi and Sugeno's first-order function (120). *Consequent parameters* {*p*_*i*_, *q*_*i*_, *r*_*i*_} in the function are determined during the training process. Lastly, one single node computes the overall output as the summation of all incoming signals ($\underset{i}{\sum }{\bar{w}}_{i}f_{i}=\frac{\underset{i}{\sum }w_{i}f_{i}}{\underset{i}{\sum }w_{i}}$). Güler and Übeyli executed a detailed classification between set A (healthy volunteer, eyes open), set B (healthy volunteer, eyes closed), set C (seizure-free intervals of five parents from hippocampal formation of opposite hemisphere), set D (seizure-free intervals of five patients from epileptogenic zone), and set E (epileptic seizure segments) using ANFIS and got the classification accuracy 98.68% (95).

##### 3.1.2.4. Radial basis function neural network (RBFNN)

*RBFNN* is feed-forward like MLPNN but has only one hidden layer with a non-linear radial basis function (RBF) in Figure 6B (119). RBF is a real-valued function whose value depends only on the distance from the origin. RBFNN has the advantages of a simple topological structure, its locally tuned neurons, and fast learning compared to MLPNN. Aslan et al. (96) compared MLPNN with RBFNN. In the case of MLPNN, 18 out of 204 focal seizure samples were classified as a generalized seizure (8.8% error rate for focal seizure), and 9 out of 47 generalized seizure samples were classified as a focal seizure (19.1% error rate for generalized seizure). RBFNN, on the other hand, showed 3.4% and 10.6% error rate for focal and generalized seizures, respectively. However, RBFNN requires to set correct initial states. Therefore, many seizure classification papers have focused on MLPNN.

### 3.2. Non-feature Based Design

According to the symptoms of seizures, various types of signal patterns appear, and it is difficult to understand all of them with specific features. Thus, no existing hand-crafted features appear universally applicable so far (33). Deep learning methods can analyze the EEG signal and learn related characteristics automatically in a supervised learning framework (121). Although there are existing works that use the classification methods described as feature-based (20, 98), we summarize these techniques in terms of non-feature based design.

#### 3.2.1. Convolutional Neural Network (CNN)

*CNN* takes the raw image data and calculates the convolution by iterating over the input data according to the filter size specified to extract the feature of the data. The shape of output data changes depending on filter size, stride, padding, max-pooling size, and so on. The classifier can perform supervised learning by matching the output data and answer classes. CNN, with its high recognition performance in medical images (122), can be as good as an epileptologist in classifying seizures by analyzing EEG plot images as being observed by Emami et al. (33). In their work, they applied CNN to long-term EEG that included epileptic seizure states. In particular, EEG data were divided into short segments based on a given time window (ranging from 0.5–10 s) and converted into EEG plot images (224 × 224 pixels), each of which was classified by CNN as seizure or non-seizure. They used VGG-16 (123) because small size convolution filters (3 × 3) are capable of detecting small EEG waves. VGG-16 is also computationally efficient and can handle non-stationary objectives. This work is meaningful because the study is the first comprehensive attempt to evaluate EEG as plot images. However, the median true positive rate of CNN 74% is still low, so we cannot use this classifier for real patients.

#### 3.2.2. Recurrent Neural Network (RNN)

**Figure 7:**
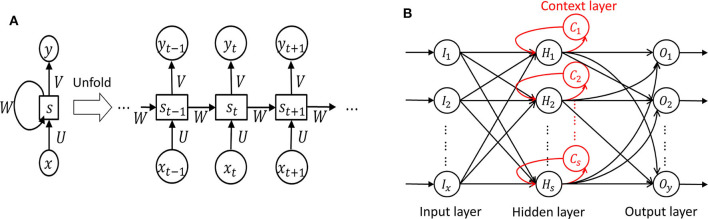
Recurrent neural network (124). **(A)** Unfolded basic. **(B)** Elman network.

In *RNN*, in which a network's output state depends on an arbitrary number of previous inputs like Figure 7A. However, RNN has not been widely used in applications due to the lack of an efficient and universal training method (124). Other attempts have been made to overcome these limitations. Srinivasan et al. (20) used a special type of RNN as Elman network (EN) to detect epileptic seizures. An EN has the additional set called “context layer” as shown in Figure 7B. The hidden layer is connected to these context units. Kumar et al. (97) incorporated recurrent EN and RBFNN to detect epileptic seizures with the wavelet entropy features. They showed 99.75 and 94.5% accuracy for detecting normal vs. epileptic seizures and interictal focal seizures, respectively.

### 3.3. Seizure Quantification

Biosignal quantification is necessary to make the correlations between biosignals and actual seizures more accurate (125). Adeli et al. (126) utilized the correlation dimension (CD, representing system complexity) and the largest Lyapunov exponent (LLE, representing system chaoticity) to quantify the nonlinear dynamics of the original EEGs. They analyzed three groups: group H (healthy subjects), group E (epileptic subjects during a seizure-free interval), and group S (epileptic subjects during the seizure). For the CD values from the band-limited EEGs (0–60 Hz), group S (5.3) differs from the other two groups H (6.9) and E (6.7). For the LLE values, group H (0.089), group E (0.041), and group S (0.070) differ from each other. CD and LLE have shown the possibility of being used for classification. However, to the best of our knowledge, there is no concrete explanation between the biosignal and the severity of symptoms.

## 4. Experimental Non-invasive Anti-seizure Treatments

We have discussed the physiological signals related to seizure as well as how to use these signals to monitor and detect seizures. The next logical step is to build a system to reduce the impact of seizure. In this section, we discuss different non-invasive brain stimulation methods that can potentially be used for seizure therapy. While we try to describe the detailed specifications, working principles, advantages, and disadvantages of different brain stimulation techniques, we leave the discussions on how to design a proper seizure therapy for future works. In particular, we discuss in detail recent non-invasive brain stimulation efforts on Transcranial Magnetic Stimulation (TMS), Transcranial Direct Current Stimulation (tDCS) (10), and Transcranial Focused Ultrasound Stimulation (tFUS) (40, 127). Since Vagus Nerve Stimulation (VNS) overlaps tDCS in terms of electrical stimulation methods and was previously introduced primarily as invasive stimulation, it was not included in the larger category. However, recently, invasive VNS therapy for drug-resistant epilepsy patients received FDA approval^7^. VNS is also a promising seizure therapy method.

### 4.1. Transcranial Magnetic Stimulation

**Figure 8:**
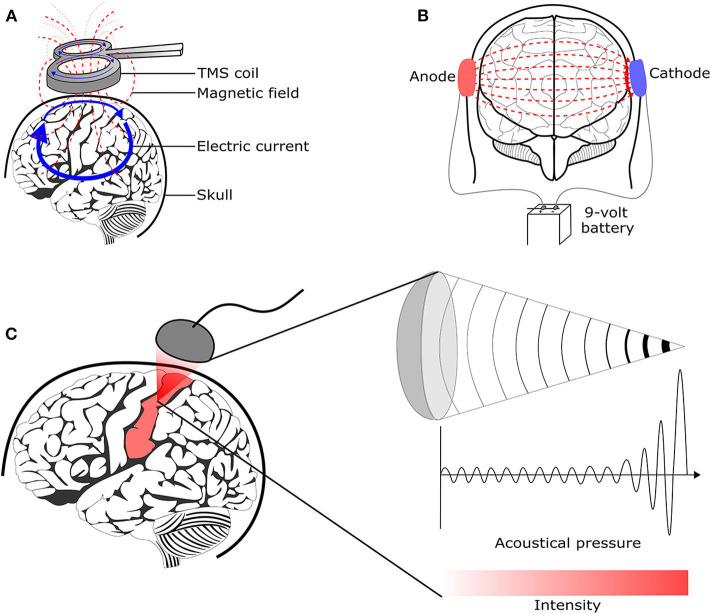
Brain stimulation methods to reduce the seizure symptoms. **(A)** Transcranial magnetic stimulation (128) **(B)** Transcranial direct current stimulation (129) **(C)** Transcranial focused ultrasound stimulation (130).

**Figure 9:**
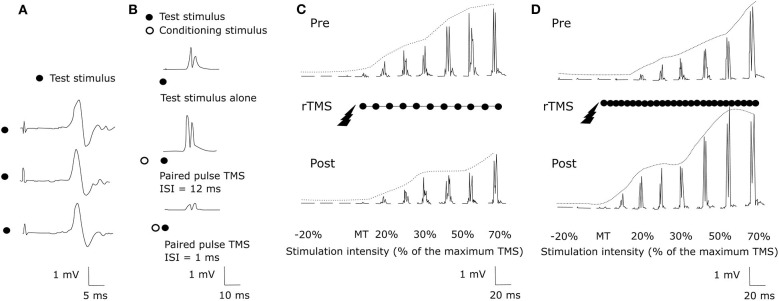
TMS methods (131). **(A)** spTMS **(B)** ppTMS **(C)** low frequency rTMS **(D)** high frequency rTMS.

Transcranial Magnetic Stimulation (TMS) uses the principle of electromagnetic induction to focus induced current in the brain, as shown in Figure 8A (37). Strong electric currents, circulating within a coil resting on the scalp, generate short and intense magnetic fields. These magnetic fields penetrate human tissue painlessly and induce electric currents that can depolarize neurons or their axons in the brain (38). TMS techniques include single-pulse TMS (spTMS), paired-pulse TMS (ppTMS), and repetitive TMS (rTMS), as shown in Figure 9 (131). In general, single- and paired-pulse TMS are used to verify brain functions, and rTMS induces changes in brain activity that can last beyond the stimulation period (132). While spTMS and ppTMS were reported to induce unexpected seizures during multiple experiments (133), rTMS is currently a better and safer approach for seizure therapy.

rTMS stimulates a single scalp site repeatedly and modulates cortical excitability. Figures 9C,D show examples tested before and after an rTMS regime. It consists of a long pattern of low (1 Hz) or high (20 Hz) frequency rTMS delivered to the left hemisphere's primary motor cortex during 28 min (131, 134). rTMS has greater effects than single-pulse TMS but also has the potential to cause seizures (38). The FDA cleared an rTMS device as a treatment to alleviate symptoms of mildly treatment-resistant depression^8^. It shows the possibility of rTMS as a treatment for relieving various symptoms.

**Figure 10:**
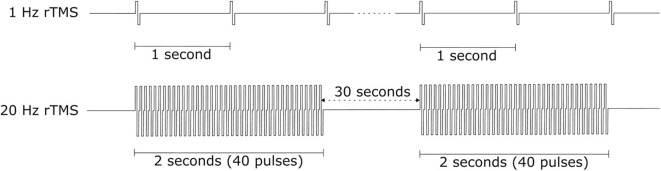
rTMS protocol example.

The effect of rTMS depends on the stimulation frequency, intensity, number of trains, inter-train interval, and number of sessions (135). We exclude the stimulus location element because it is a factor that varies depending on the symptom. The number of pulses per second of rTMS trains typically ranges between 1 and 50 Hz. One hertz paradigms are commonly applied continuously for several minutes, while higher frequency paradigms are applied in a patterned fashion like Figure 10 (136). Low-frequency rTMS produces a transient reduction in cortical excitability. High-frequency rTMS produces a local increase in cortical excitability and increases in MEP size (137). Specifically, this transient reduction effect of Low-frequency rTMS occurs in the motor cortex (138) and in the occipital cortex (139). High-frequency rTMS can improve cognitive processing to the dorsolateral prefrontal cortex (140). To compare low and high-frequency rTMS, Speer et al. (141) showed that 1-Hz rTMS was associated only with decreases in absolute regional cerebral blood flow (rCBF), while twenty-Hertz rTMS over the left prefrontal cortex was associated only with increases in rCBF.

The stimulation intensity is usually expressed as a percentage of MT. The MT is usually determined before each session by applying the TMS coil over the primary motor cortex (135). Pulse trains are the typical form to use rTMS. If TMS stimulates the brain continuously, it can increase the possibility of generating a seizure (142) and cause heating of the electrodes. Flitman et al. (143) reported the occurrence of focal to bilateral tonic-clonic seizure in one subject after three consecutive stimulated trials with 20% above MT and pulse train lasted 750 ms at 15 Hz. Dobek et al. (144) also found 25 reports of rTMS-induced seizures in their review. Therefore, we should follow the safety guidelines for rTMS (145). Based on the international workshop on the safety of rTMS in 1996, Wassermann et al. (146) introduced the guideline for the use of rTMS: at least 5 s intervals between 20 Hz trains with intensities of up to 1.1x the MEP threshold. A longer interval is necessary for the case of higher frequencies and intensities. Bae et al. investigated the risk of seizures associated with rTMS in patients with epilepsy and reported that only 4 of 280 patients experienced seizures during or after rTMS (147).

In animal studies, low-frequency rTMS (1,000 pulses at 0.5 Hz) decreased susceptibility to pentylenetetrazol-induced seizures in rats (148). Rotenberg et al. (149) suppressed seizures in rats injected with the kainic acid using EEG-guided 0.5 and 0.75 Hz rTMS, but 0.25 Hz rTMS was not effective. In human studies, 0.3 Hz low-frequency rTMS decreased interictal EEG epileptiform abnormalities in one-third of drug-resistant epilepsy patients but was not better than a placebo for seizure reduction (150). Instead, Cincotta et al. (151) suggested that 0.3 Hz rTMS produces a relatively long-lasting enhancement of the inhibitory mechanisms responsible for the cortical silent period. Low-frequency rTMS decreased the number of seizures in patients with focal neocortical epilepsy (35) and refractory epilepsy (152).

### 4.2. Transcranial Direct Current Stimulation

**Figure 11:**
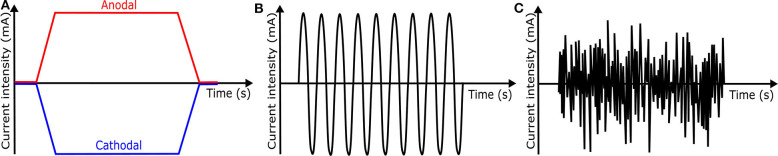
Waveforms of different tES techniques. **(A)** tDCS waveform **(B)** tACS waveform **(C)** tRNS waveform.

tDCS is one of transcranial electrical stimulation (tES) methods and applies low-amplitude direct currents via scalp electrodes and penetrate the skull to enter the brain, as shown in Figure 8B (37). The principal difference between tDCS and other tES techniques is the waveform to the brain, like Figure 11. tDCS is the only class of neuromodulation technique that delivers a sustained direct current (DC) like Figure 11A (39). Transcranial alternating current stimulation (tACS) has a variety of stimulation with different frequencies (1–45 Hz), like Figure 11B (153). tACS enables the study of causal links between brain rhythms and specific aspects of behavior. Transcranial random noise stimulation (tRNS) follows a white-noise band-limited waveform (0.1–640 Hz) like Figure 11C (154). tRNS focuses on the link between behavior and frequency-specific noise inherent in neural processing (127). tACS and tRNS are usually used to identify or compare frequency-specific characteristics. They are not actively used as therapeutic methods to obtain actual effects compared to tDCS. Besides, tDCS is easier to use, cheaper, and more tolerable than TMS. However, tDCS is still an experimental form of brain stimulation and is not an FDA-approved treatment^9^. tDCS does not induce neuronal action potentials because static fields do not yield the rapid depolarization required to produce action potentials in neural membranes (155). Thus, it is a pure neuromodulatory intervention. tDCS could modulate cortical excitation and cortical inhibition by anodal polarity and cathodal polarity, respectively. By varying the current intensity and duration, the strength and duration of the after-effects could be controlled.

#### 4.2.1. Factors

The effect of tDCS depends on current density, stimulation duration, the orientation of the electric field (the electrodes' positions and polarity), electrode configuration (material and size), the patient, deep of the target, and intensity of the current (156, 157). Long-lasting stimulation largely influenced the durability of after-effects to humans (158). tDCS protocols should specify electrode position and current direction because these elements cause different stimulation results. The electrodes for tDCS are usually a pair of electrodes covered by sponges filled with a contact medium such as NaCl solution or conductive cream (155). For the electrode size, although large electrodes expand the area of the excitability modification, small electrodes are better to increase tDCS focality (155).

#### 4.2.2. Case Studies

Cathodal tDCS leads to a reduction of cortical excitability by decreasing the neuronal firing rate and inducing long-term depression (LTD) of neuronal excitability (159). In animal studies, cathodal tDCS at 100 μA for 60 min resulted in a duration of more than 2 h with an increasing threshold of focal onset seizure activity, while anodal tDCS had no significant effect on TLS in the rat (160). In human studies, cathodal tDCS may be effective to reduce seizures' frequency as shown in Table 3. Most tDCS related works applied 1–2 mA cathodal tDCS for 20–60 min. Yook et al. (174) placed a tDCS cathode at the midpoint of P4 and T4, where the 11-year-old female seizure patient showed the abnormal EEG wave. After 2 mA cathodal tDCS for 20 min, the number of seizure occurrence and the duration of each seizure episode were reduced.

**Table 3:** Experimental non-invasive neuromodulation treatments for epilepsy [Reviews for readers who want to know more about neuromodulation treatments: (161–163)].

**References**	**Brain stimulation^**a**^**	**Subjects**	**Seizure type^**b**^ (43)**	**Experiment^**c**^**	**Result**
(164)	Low frequency rTMS	Human	FIAS	rTMS was done on consecutive days to nine patients by means of a round coil (9 cm diameter). Four weeks before and after the treatment, patients were requested to record every definite seizure or any seizure-like event	Although in two patients a partial seizure occurred directly after rTMS, in none of the patients did seizures per week increase under rTMS
(165)	Low frequency rTMS	Human	Focal motor seizures (Focal myoclonic seizures and FIAS)	EMG records of a drug-resistant epilepsy patient were recorded for 15 min before and after 1 Hz rTMS	Before rTMS, 60 muscle jerks >100μ*V* were detected. After intervention with rTMS, only 20 muscle jerks accomplished the chosen criterion of >100μ*V*
(150)	Low frequency rTMS	Human	Focal aware (7), FIAS (14), FTCS (18), Others (4)	The stimulus frequency was 0.3 Hz. One thousand stimuli per day were given for 5 consecutive days with a round coil at the vertex	0.3 Hz low-frequency rTMS decreased interictal EEG epileptiform abnormalities in one-third of drug-resistant epilepsy patients, but was not better than placebo for seizure reduction
(35)	Low frequency rTMS	Human	Focal neocortical epilepsy	rTMS with 900 pulse, intensity of 120% motor resting threshold and 0.5 Hz frequency was used to 12 patients	The mean seizure frequency was 2.25, 0.66, and 1.14 seizures per week before, during, and after rTMS, respectively
(152)	Low frequency rTMS	Human	Focal onset seizures	Twenty-one patients with malformations of cortical development and refractory epilepsy underwent five consecutive sessions of low-frequency rTMS	rTMS significantly decreased the number of seizures in the active compared with sham rTMS group (*p* < 0.0001), and this effect lasted for at least 2 months
(166)	Low frequency rTMS	Human	Focal aware (4), FIAS (2), FTCS (54), Others (20)	Sixty patients were randomly divided into two groups by stimulation intensity: 90% (Group 1) or 20% (Group 2) of rMT	In Group 1, the effects of rTMS on IED of 60 min were 75.1, 23.1, and 33.6 before, during, and after rTMS, respectively. However, Group 2 doesn't show any difference by rTMS
(167)	Cathodal tDCS	Human	Focal onset seizures	A single treatment with 1 mA cathodal tDCS for 20 min over the seizure focus and anode on the contralateral shoulder (36 children, 27 in active and 9 in sham group)	Active tDCS treatment was associated with significant reductions in epileptic discharge frequency in both 24 (50.3%) and 48 (57.6%) hours after the treatment
(36)	Cathodal tDCS	Human	FIAS	Two patients with drug-resistant FIAS received the sham or the real tDCS treatment on the 8th and 22th days	Both patients underwent a consistent reduction of the frequency of the seizures, 70 and 50%, respectively
(168)	Cathodal tDCS	Human	Focal onset seizures	Twelve patients received the modulated cathodal tDCS (30 min/2 mA × 3 days) and the sham stimulation	The mean seizure frequency was 10.58 ± 9.91 at the baseline and decreased to 1.67 ± 2.50 after cathodal tDCS
(169)	Cathodal tDCS	Human	FIAS	Ten patients with drug-resistant TLE received the sham or the real tDCS treatment on day 8 and day 38	tDCS reduced the percent weekly seizure frequency more than sham stimulation (−71 ± 33% and 25+−125%)
(170)	Cathodal tDCS	Human	Focal or generalized onset seizures	Group 1: 30 min/2 mA × 3 days, *n* = 12 Group 2: 30 min/2 mA × 5 days, *n* = 8 Group 3: placebo, *n* = 8	The mean reduction of seizure frequency in both active groups was significantly higher than placebo (Group 1: −43.4%, Group 2: −54.6%, Group 3: −6.25%)
(171)	Cathodal HD-tDCS	Human	Focal or generalized onset seizures	20-min sessions of 2 mA HD-tDCS were applied for 10 consecutive days to ten adult patients	Changes of epileptiform discharges and mean seizure frequency caused by HD-tDCS were not statistically significant (*p* > 0.05)
(172)	tFUS	Cat	Focal onset seizures	Experimental focal epilepsy was induced in thirty-six cats by subcortical injection of 0.1 ml of alumina cream. Then 15 -> surgical removal, 12 -> tFUS, and 9 -> medication	Surgery: 6 died, 8 seizure-free 12 weeks, 1 recurred tFUS: 1 died, 9 seizure-free for 12 weeks, 2 recurred Medication: 9 died
(173)	tFUS	Rat	Pentyleneterazol-induced seizures	An ultrasound transducer operating at a fundamental frequency of 690 KHz was applied twice for 3 min each. Group 1: tFUS sonication after PTZ administration, *n* = 9 Group 2: without sonication after PTZ administration, n = 9	Group 1: After the second sonication, the number of EEG bursts in the FUS-treated rat was reduced clearly compared to that of the first sonication. Group 2: The number of EEG bursts was kept during the entire monitoring period.

a *TMS, Transcanial magnetic stimulation; rTMS, Repetitive TMS; tDCS, Transcranial direct current stimulation; HD-tDCS, High definition tDCS; tFUS, Transcranial focused ultrasound stimulation*.

b *FIAS, Focal onset impaired awareness seizures; GTCS, Generalized tonic-clonic seizures; FTCS, Focal to bilateral tonic-clonic seizures*.

c *rMT, resting motor threshold*.

#### 4.2.3. Safety

Several metrics, including current density, duration, and the charge, should be controlled carefully to prevent serious adverse effects. Bikson et al. (175) investigated the related papers for the safety of tDCS and offered the criterion of tDCS protocol: current density (6.3−13 *A*/*m*^2^) from the animal models, and others (≤ 40 *min*, ≤ 4 *mA*, ≤ 7.2 *Coulombs*) from the human trials. Under this condition, there were no reports of severe side effects across over 33, 200 sessions and 1,000 subjects (175).

#### 4.2.4. Deep Brain Stimulation of tDCS

tDCS can only directly stimulate in cortical regions. To overcome this limitation, Grossman et al. (176) suggested a new protocol called temporal interference non-invasive brain stimulation (TI-NIBS). TI delivers multiple electric fields to the brain at frequencies which are too high to recruit neural firing. These multiple electric fields differ by a frequency within the dynamic range of neural firing. They applied TI-NIBS to a living mouse brain and demonstrated the effects of TI-NIBS by stimulating neurons in subcortical structures (176). While the current experiment has not yet been applied to humans, we believe this is one of the most potential approaches for seizure therapy in the future due to its capability of providing high spatial and temporal resolution. In addition, since the stimulation is only effective at the locations where all the beams are constructive, the beams may not harm the brain cells that are not located in the targeted areas.

### 4.3. Transcranial Focused Ultrasound Stimulation

tFUS is emerging as a method to improve the relatively low degree of spatial locality offered by TMS and tDCS (127). It is important because a low degree of spatial locality leads to modulating neuronal activity not only in the target but also in surrounding circuits. tFUS uses acoustic energy to stimulate the brain like Figure 8C. tFUS can both excite and suppress brain neuronal activity and has millimeter spatial resolutions (177). In 1988, Colemann and Lizzi developed the Sonocare CST-100, which is the first high intensity focused ultrasound and received the FDA pre-market approval^10^. The device was designed for the treatment of glaucoma.

#### 4.3.1. Factors

There are some factors to control the effect of tFUS: acoustic frequencies, intensities, and modes of transmission (178). First, Ultrasound (US) has a frequency above 20 kHz. Most medical US frequency range is between 1 and 15 MHz, and therapeutic US application operates around 1 MHz or less (179). Second, therapeutic US can be divided into low power (<0.5 *Wcm*^−2^) or high power (>100 *Wcm*^−2^) depending on the acoustic intensity (178). Low power intensity US is used in physiotherapy, non-thermal actions, and so on, whereas high power intensity US is used in lithotripsy and the thermal ablation of tissue (180). The therapeutic US usually has low power intensity. Lower energy US increased the action potential, while higher energy US reduced the action potential due to the ultrasonic thermal effects. Third, the US has two modes of transmission: continuous wave (CW) and pulsed wave (PW) (178). CW stimuli are more effective than PW stimuli in eliciting responses (181). Therapeutic US is usually delivered as CW or long pulse exposures (180).

#### 4.3.2. Current Progress

In animal studies, Manlapaz et al. (172) reported ultrasonic irradiation relieved the seizures of cats. They compared fifteen cats treated by surgical removal of the epileptogenic focus with twelve cats treated by ultrasonic irradiation. The ultrasonic approach showed less postoperative complications than those of surgery. Min et al. (173) injected pentylenetetrazol to rats to induce acute epilepsy and applied tFUS to the rat's brain twice for 3 min. Epileptic EEG signals of the rats decreased visibly after tFUS compared to the other group that did not receive any tFUS. In human studies, to the best of our knowledge, there are no existing works to handle the relationship between seizure and tFUS. Instead, we look at studies that relate the human brain to tFUS. Legon et al. (40) evaluated if tFUS is capable of modulating brain activity in the human primary somatosensory cortex. From the experiment, tFUS remarkably reduced the amplitude of somatosensory evoked potential. Lee et al. (182) reported tFUS of the primary visual cortex. The tFUS induced activation both from the sonicated brain area and from the visual or cognitive network regions. However, tFUS beam might potentially harm brain cells when it passes through them.

#### 4.3.3. Safety

It is difficult to establish tFUS protocol for safety now because there is no enough medical data about tFUS. Although Legon et al. (183) applied low power tFUS to 120 participants who did not report any neurological impairment and reported that none of the participants experienced serious adverse effects, it does not prove the safety of tFUS. This is because ultrasound at high intensities can cause irreversible tissue damage (40). The safety protocol could be established from a variety of tFUS related experiments. US studies are necessary to be conducted on primate brains such as monkeys having a skull with similar thickness and size to that of humans (184).

## 5. Potential Research Directions

We have discussed state-of-the-arts sensing and stimulation technologies that are suitable for seizure monitoring and therapy. In this section, we present potential research directions that require more attention to building a robust, wearable, safe, sensing, and stimulation systems.

### 5.1. Robust and Wearable Seizure Detection System

#### 5.1.1. Monitoring Seizures From the Brain With High Resolution

Current technologies only allow us to monitor the whole brain. However, we envision a more robust sensing technology that could sense precisely where the seizure signal occurs on the human brain. The future sensor can be implemented as an array of electrodes to form a beam-forming receiver to capture only the brain area of interest. It will improve the performance of the sensing system by efficiently removing the interference signals from many non-related brain areas. TI-NIBS (176) design can be considered as the closest reference.

#### 5.1.2. Improving Seizure Quantification

Most existing seizure detection systems have only focused on differentiating between seizure and non-seizure. Therefore, we could not find a concrete explanation between the biosignal and the severity of symptoms from the related works. The biggest problem is that there are no clear criteria for quantifying seizures, and it is difficult to obtain the ground truth data. Once reasonable standards are established, and researchers' consent is received, the seizure quantification will be applied to the seizure detection system quickly.

#### 5.1.3. Making Seizure Monitoring System Become Wearable

We predict the core location of recording EEG signals will be around the ear. The reason is that the device around the ear can still acquire clear EEG signals and does not restrict the user's mobility. Dry electrodes could be applied to the ear-cover part (185) of the device to improve usability. Different biosignals, including EOG, EMG, and EDA, also could be detected with EEG signals around the ear. A headband with EEG electrodes is also used to detect seizures from the frontal lobe or other locations which could not be detected by seizure detection devices around the ear. The wearable devices can deliver biosignals to a smartphone through communication technology. The application for seizure detection on the smartphone will extract features and classify seizure types.

### 5.2. Safe, Accurate, and Reliable Seizure Stimulation

#### 5.2.1. High Spacial Resolution Stimulation

The stimulation device needs to localize the target area of the brain and stimulate it accurately. Existing TMS and tDCS have a low degree of spatial locality. We introduced several approaches to solve this problem. Hesed coil design of TMS attaches several strips on the specific part of the head intensively with wires that induce stimulation in the desired direction (186). TI-NIBS, as an alternative of tDCS, delivers multiple electric fields to the brain at frequencies that are too high to recruit neural firing (176). In addition, new tDCS algorithms allow a better focal treatment using multi-target electrodes and smaller electrodes in High-Definition tDCS (HD-tDCS) (187). These approaches are likely to advance to deep brain stimulation and require additional studies because they are in the proposal stage.

#### 5.2.2. Safe and Reliable Stimulation Device

Safety is the most crucial aspect of designing the stimulation system. A practical system should prioritize the safety aspect, for example, long term and short term side effects. Unlike TMS achieving some degree of safety, tDCS and tFUS are necessary to establish the safety protocol. Antal et al. (188) introduce detail information about the safety of tDCS, including long stimulation duration, montages with (multiple) small electrodes, and limiting the maximum current. Although there are a lot of works for tDCS, tDCS still only happens in the lab environment and is not an FDA-approved treatment solutions^9^. Rather, as another electrical stimulation approach, non-invasive VNS therapy for drug-resistant epilepsy patients received FDA approval^7^. In the near future, we believe the establishment of a tDCS safety protocol for humans by integrating its new experiment with existing work. Meanwhile, there are a few related works about tFUS. Many tests for tFUS are necessary before establishing a safety protocol.

#### 5.2.3. Making Seizure Therapy System Become Wearable

We believe tDCS could be integrated with the seizure detection system, especially around the ear. tDCS applies low-amplitude direct currents via two scalp electrodes like Figure 8B. Each scalp electrode could be connected to the surrounding area of the left ear and right ear, respectively. When designing a circuit, we need to consider the difference between the battery used for the existing seizure detection device and that used for tDCS. Unless a new design is available, it seems difficult to create a wearable device that incorporates TMS or tFUS and a seizure detection device. The fact that both brain stimulation methods are applied with a small gap between the brain and the device makes it difficult to make a wearable device.

### 5.3. An Integrated Sensing and Stimulation System

Even when the seizure monitoring and stimulation systems are reliable, there are many challenges remaining in integrating these two components to produce a reliable integrated system in a wearable form.

## 6. Conclusion

. In this paper, we systematically categorized recent efforts on building seizure monitoring, detection, and therapy systems. We explained the overall systems and components that can be used to monitor the reliable physiological signals of seizures. We presented different techniques for extracting physiological seizure signals from the noises. Then, we discussed in detail recent effort on classifying/detecting seizure events using machine learning and deep learning. Next, we presented different seizure therapy techniques, including TMS, tDCS, and tFUS. Last but not least, we discussed potential future research directions on building a wearable seizure detection and therapy system based on our experience in building comprehensive health solutions.

## Author Contributions

TK surveyed and wrote all parts of the manuscript. TK and PN discussed and wrote Lesson Learned and section 6. TK, PN, NP, NB, HT, SH, and TV revised the manuscript.

## Conflict of Interest

The authors declare that the research was conducted in the absence of any commercial or financial relationships that could be construed as a potential conflict of interest.

## Footnotes

^1^https://www.epilepsysociety.org.uk/epileptic-seizures#.XsfrI2hKiHs

^2^https://www.safetysystemsdistribution.co.uk/emfit-tonic-clonic-seizure-monitor-basic/

^3^https://medpage-ltd.com/epileptic-tonic-clonic-seizure-alarm-MP5

^4^https://smart-monitor.com/about-smartwatch-inspyre-by-smart-monitor/

^5^https://www.bioserenity.com

^6^https://www.iec.ch/worldplugs/list_bylocation.htm

^7^https://www.mobihealthnews.com/content/fda-approves-sentiva-nerve-stimulation-device-epilepsy-therapy

^8^https://www.fisherwallace.com/

^9^https://www.hopkinsmedicine.org

^10^https://www.fusfoundation.org/the-technology/timeline-of-focused-ultrasound
